# Dissolvable Microneedle Delivery of a Replication-Deficient Orthopoxvirus Vaccine: Formulation Screening and Immunogenicity Evaluation for Monkeypox Prevention

**DOI:** 10.3390/vaccines14030276

**Published:** 2026-03-20

**Authors:** Bin Wang, Kehui Wang, Zhiyao Xu, Weihua Liu, Xianhuang Li, Linhao Li, Renhui Zhou, Xingyue Du, Jin Jin, Yaqing Xu, Rihui Qin, Xiong Liu, Dayang Zou, Wei Liu

**Affiliations:** 1 Department of Epidemiology and Biostatistics, School of Public Health, Anhui Medical University, Hefei 230032, China; w179936503@163.com (B.W.); 2445010447@stu.ahmu.edu.cn (Z.X.); 2 Chinese PLA Center for Disease Control and Prevention, Beijing 100071, China; ake1999@163.com (K.W.); liuweihua@cmu.edu.cn (W.L.); lixianhuang@tju.edu.cn (X.L.); llh962037145@163.com (L.L.); zhou7633@foxmail.com (R.Z.); liudong19910828@sina.com (X.D.); jinjin9307@163.com (J.J.); 19155876242@163.com (Y.X.); 18801398536@163.com (R.Q.); liuxiong714@163.com (X.L.); 3 Department of Health Statistics, School of Public Health, China Medical University, Shenyang 110122, China

**Keywords:** non-replicating Tiantan vaccinia virus (NTV), dissolving microneedle (DMN), thermostability, vaccine formulation, stabilization mechanism

## Abstract

**Background:** The global spread of monkeypox virus (MPXV) highlights an urgent need for thermostable and easily administrable vaccines. Current orthopoxvirus vaccines are limited by cold-chain dependence and inconvenient injection-based delivery. **Objectives:** This study aimed to develop a dissolvable microneedle (DMN) vaccine against monkeypox based on a replication-deficient orthopoxvirus platform, through systematic formulation screening, stabilization mechanism exploration, and rigorous in vivo immunogenicity evaluation. **Methods:** A film-based approach was adopted for efficient, high-throughput formulation screening and thermostability assessment. NTV was mixed with excipients and dried into solid films. Stability was monitored via RT-qPCR after storage at 4 °C to 40 °C. The lead formulation was physically characterized, then used to fabricate MVA-BN-loaded DMN patches, which were further evaluated for in vivo immunogenicity via immunization in *BALB/c* mice. **Results:** The optimal formulation F2 (containing dextran, L-threonine, and BSA/HSA) showed a potency loss of only ~1 log_10_ after 2 months at 25 °C, and <1 log_10_ loss after 1 week at 37 °C. SEM revealed a porous virus-entrapment morphology, and FTIR indicated enhanced hydrogen bonding between the virus and the dextran matrix. The formulation was successfully manufactured into DMNs that dissolved within 5 min. In mice, these DMNs elicited robust MPXV-specific IgG and neutralizing antibody responses, with immunogenicity comparable to that induced by conventional intramuscular injection. **Conclusions:** This study successfully established a thermostable formulation and dissolvable microneedle delivery platform for replication-deficient orthopoxvirus vaccines against monkeypox. The optimized DMN vaccine induced robust MPXV-specific immune responses in mice with immunogenicity comparable to intramuscular injection, addressing the core limitations of current vaccines and providing a promising solution for monkeypox prevention.

## 1. Introduction

*Monkeypox virus* (MPXV), a member of the genus *Orthopoxvirus* within the subfamily *Chordopoxvirinae* and family *Poxviridae*, is an enveloped, linear double-stranded DNA virus [1,2]. Its core genomic region encodes essential enzymes and structural proteins, sharing 96.3% sequence similarity with the *variola virus*, the causative agent of smallpox [3,4]. Although MPXV infection exhibits a lower case-fatality rate than smallpox, it leads to substantial dermal destruction and frequently results in permanent scarring [5]. Prior smallpox vaccination offers partial cross-protection; however, the discontinuation of global smallpox vaccination campaigns has resulted in a marked decline in population-wide immunity against orthopoxviruses [6,7,8]. Current epidemiological evidence suggests that this expanded immunologically naïve population is a key factor driving the recent increase in monkeypox incidence.

Vaccination remains one of the most effective strategies for infectious disease prevention [9]. Nevertheless, no MPXV-specific vaccines have been approved worldwide to date [10]. Consequently, the World Health Organization (WHO) recommends three smallpox vaccines—LC16m8 (KM Biologics), MVA-BN (Bavarian Nordic; a replication-deficient attenuated live vaccine), and ACAM2000 (Emergent BioSolutions; a replication-competent attenuated live vaccine)—for individuals at risk of MPXV infection [11]. However, these vaccines present notable limitations: MVA-BN (e.g., Imvamune^®^), as a lyophilized attenuated formulation, requires stringent cold-chain conditions that increase logistical costs and complicate deployment, especially in resource-limited settings. Its multi-dose subcutaneous administration regimen also adversely affects patient compliance [12]. In contrast, both LC16m8 and ACAM2000 rely on bifurcated needle scarification—an invasive and technically demanding procedure associated with inconsistent dosing, potential infection risks, and a high dependency on trained healthcare workers, thereby hindering widespread use in primary care contexts [13,14]. Non-replicating Tiantan vaccinia virus (NTV), a replication-defective orthopoxvirus that retains full orthopoxvirus immunogenicity with abrogated replication capacity in human cells [15], has been approved for clinical trials of monkeypox prevention in China, representing a promising candidate strain for the development of novel orthopoxvirus vaccines.

Emerging microarray patch (MAP) technologies offer a promising alternative by simplifying vaccine administration and potentially enabling dose-sparing through intradermal delivery, which leverages the skin’s immunogenicity [16]. Among various MAP designs, dissolving microneedles (DMNs) present distinct advantages for vaccine formulation [17]. DMNs are fabricated by incorporating antigens into a matrix of biocompatible, water-soluble materials, which are dried and solidified into microneedle arrays using diverse manufacturing techniques [18]. These MAPs consist of numerous micron-scale projections that penetrate the stratum corneum upon application. Subsequent dissolution in the skin releases the vaccine payload directly into the epidermal and dermal layers, facilitating robust immune activation [19]. Preclinical studies and early-phase clinical trials evaluating various vaccine antigens have demonstrated the promising potential of DMN-based delivery systems [20,21]. Moreover, MAP formulations designed for low- and middle-income countries (LMICs) eliminate the need for needles and syringes, enabling simpler administration (potentially by minimally trained personnel or through self-administration), reducing transportation costs due to their compact size, and improving stability during storage and shipping [22,23]. These benefits collectively could substantially increase vaccination coverage in LMICs and help curb the spread of infectious diseases [24].

Building on a previously established methodology for stabilizing vaccine antigens for microarray patch (MAP) delivery, including the successful formulation of trivalent inactivated polio vaccine (tIPV) [25] and live attenuated measles–rubella (MeRu) vaccine [26], we extend this solid-state stabilization strategy herein to the development of a dissolvable microneedle (DMN) vaccine for monkeypox prevention. The core objective of this work is to establish a DMN delivery platform for replication-deficient orthopoxvirus vaccines, through formulation optimization and in vivo immunogenicity assessment for monkeypox prevention. Given the highly conserved antigen profile and MPXV cross-protective immunity between replication-deficient orthopoxviruses, we selected the globally licensed MVA-BN strain as the validation strain to evaluate the in vivo immunogenicity of the DMN vaccine fabricated with our optimized formulation.

## 2. Materials and Methods

### 2.1. Materials

NTV strain was provided by Beijing Institute of Biological Products (BIBP, Beijing, China) under MTA #BIBP-MTA202403304. The viral titer is 5 × 10^8^ CCID_50_/mL, stored at −80 °C as frozen bulk. MVA-BN strain was gifted by Shanghai Institute of Biological Products Co., Ltd. (SIBP, Shanghai, China), with a viral titer of 5 × 10^8^ CCID_50_/mL, stored at −80 °C as frozen bulk. All experiments involving live NTV and MVA-BN viruses were performed in a Biosafety Level 2 (BSL-2) laboratory in strict compliance with national biosafety regulations. The Monkeypox virus (MPXV) strain used for neutralization assays was obtained from the Wuhan Institute of Biological Products Co., Ltd. (WIBP, Wuhan, China) and handled in their BSL-3 facility. The BHK-21 cell line used in this study was obtained from Procell Life Science & Technology Co., Ltd. (Wuhan, China). The Vero E6 cell line was obtained from Acells (Shanghai, China; Cat. No. AC801). Isoflurane was obtained from ACMEC (Shanghai, China). Hyaluronic acid (HA), dextran, sucrose, and bovine serum albumin (BSA) were obtained from Macklin (Shanghai, China). Maltodextrin, hydroxyethyl starch, polyvinyl alcohol (PVA), carboxymethyl cellulose (CMC), L-arginine, L-glutamic acid, L-threonine, maltose monohydrate, D-mannitol, and D-(+)-trehalose dihydrate were purchased from Aladdin (Shanghai, China); glycinate and chitosan were also supplied by Aladdin. D-sorbitol and D-mannose were procured from Sigma-Aldrich (St. Louis, MO, USA). Polyvinylpyrrolidone (PVP) and fish skin gelatin were procured from Sigma-Aldrich. Pullulan was supplied by Solabio (Beijing, China). Hydroxypropyl methylcellulose (HPMC) was acquired from Boer (Shanghai, China). D-Galactose was purchased from Vetec (Sigma-Aldrich). Cell culture plates, including 12- and 24-well plates (Corning, NY, USA) and 96-well plates (Nest, Wuxi, China), were used in this study. Dulbecco’s Modified Eagle Medium (DMEM) and Minimum Essential Medium (MEM), both supplemented with 10% fetal bovine serum (FBS; Gibco, Thermo Fisher Scientific, Waltham, MA, USA) and 1% penicillin–streptomycin (Gibco), were obtained from Invitrogen (Thermo Fisher Scientific, Waltham, MA, USA). Phosphate-buffered saline (PBS, 10 mM) and 0.25% Trypsin–EDTA solution were purchased from Gibco. For the PRNT_50_ assay, the 4% neutral buffered formaldehyde fixative was obtained from Servicebio (Wuhan, China; Cat. No. G1101), and the crystal violet staining solution was sourced from Solarbio (Cat. No. G1061). Human serum albumin (20%) was obtained from Chengdu Rongsheng Pharmaceutical Co., Ltd. (Chengdu, China). Cell culture flasks (25 cm^2^ and 75 cm^2^) were supplied by KIRGEN (Shanghai, China).

### 2.2. Methods

#### 2.2.1. Preparation of Excipient Stock Solutions and Virus Formulations

Concentrated stock solutions of excipients were prepared in 10 mM PBS, and the pH was adjusted to reach the target pH. These operations were performed in a biological safety cabinet to ensure aseptic conditions. NTV was initially diluted in 10 mM PBS buffer to achieve a target titer. Then, equal volumes of diluted NTV viral solution and concentrated excipient stock solution were mixed at a 1:1 volume ratio (*v*/*v*) using a micropipette. A total volume of 150 µL of the mixture, containing a theoretical viral load of approximately 2 × 10^6^ CCID_50_. The mixture was then transferred into the caps of 2 mL microcentrifuge tubes and dried under controlled conditions (either at 4 °C or 25 °C, and 20% relative humidity (RH) for 48 h or 24 h, respectively) to form solid composite films. This approach of creating dried material–virus blended films simulates the loading of virus onto microneedles, enabling high-throughput and rapid screening. A sample was considered sufficiently dried and suitable for downstream analysis if it formed a dry, handleable solid film. Specifically, after the drying process, the formulation must solidify into a cohesive, continuous film that could be easily separated from the microcentrifuge tube cap and fully rehydrated. Films that remained sticky, fragmented, or could not be completely resuspended were excluded from further analysis, as they would not yield reliable assay results. Then, the 2 mL microcentrifuge tubes were securely sealed and transferred to controlled storage environments at designated temperatures. After the designated storage period, reconstitution was performed by adding 1 mL of pre-warmed (37 °C) DMEM complete medium (supplemented with 2% fetal bovine serum, sterile-filtered) directly into each 2 mL microcentrifuge tube containing the dried composite film. The mixture was gently agitated at 25 °C for 5 min to ensure complete dissolution. Subsequently, the mixture was vortex-mixed for 15 s at 1000 rpm to homogenize the solution, and samples were centrifuged at 2000× *g* for 2 min at 25 °C to collect residual material adhering to the tube walls. The freshly reconstituted samples were immediately subjected to analytical testing without any additional storage.

#### 2.2.2. Titration of NTV Infectivity in BHK-21 Cells via CCID_50_ Assay

The infectious titer of NTV samples was determined by a 50% Cell Culture Infective Dose (CCID_50_) assay on BHK-21 cell monolayers. It is important to note that the NTV strain used is replication-deficient in human cells but remains fully permissive and replication-competent in BHK-21 cells; therefore, this cell line was suitable for all infectivity and stability assessments. Serial 10-fold dilutions of the virus were prepared in maintenance medium free of virus. Confluent monolayers of BHK-21 cells in 96-well plates were inoculated with 100 µL of each viral dilution per well. Following inoculation, the plates were incubated at 37 °C under 5% CO_2_ for 72–96 h. Cytopathic effect (CPE) was evaluated microscopically based on predefined morphological criteria, including cell rounding, detachment, and syncytia formation. The CCID_50_ titer was subsequently calculated using the Reed–Muench method.

#### 2.2.3. Quantification of NTV Infectivity by RT-qPCR and Viral Genomic DNA by qPCR

In this work, the infectivity of NTV was quantified by an RT-qPCR assay targeting viral early gene transcription, while the viral genomic load was determined by direct qPCR to assess the physical loss of viral particles. For the infectivity RT-qPCR assay, BHK-21 cell monolayers were inoculated with 50 μL of serially diluted NTV solution or reconstituted film samples, and incubated at 37 °C under 5% CO_2_ for 24 h. Total RNA was extracted using the RNA Easy Fast Tissue/Cell Total RNA Kit (DP451, Tiangen Biotech), and one-step RT-qPCR was performed to amplify the viral *E9L* gene and the endogenous reference *β-actin* gene. The primers and probe for the *E9L* gene were as follows: forward primer 5′-GAACATTTTTGGCAGAGAGAGCC-3′, reverse primer 5′-CAACTCTTAGCCGAAGCGTATGAG-3′, and FAM-labeled probe 5′-FAM-CAGGCTACCAGTTCAA-MGBNFQ-3′. The 25 μL reaction system and thermal cycling conditions were set according to the reagent manufacturer’s instructions, and the relative expression of *E9L* mRNA was calculated via the ΔΔCt method. Viral titer loss was expressed as a log_10_-transformed value relative to the freshly thawed NTV bulk stock, and the RT-qPCR signal was used as a high-throughput indicator for formulation stability screening. For viral genomic DNA quantification, total viral DNA was extracted from reconstituted samples using the TIANamp Virus DNA/RNA Kit (DP315, Tiangen Biotech, Beijing, China), and the *E9L* gene was quantified via qPCR using the same primers, probes and cycling conditions described above.

#### 2.2.4. NTV Viral Yields and Stability Evaluations

To evaluate NTV vaccine yields and stability following drying and storage in the solid state, in vitro potency values of reconstituted solid composite films NTV vaccine samples (or liquid controls) were determined using the infectivity RT-qPCR or CCID_50_ assays as described above. NTV vaccine stability was evaluated as titer (potency value) or titer loss. A liquid NTV bulk vaccine sample stored at −80 °C (frozen bulk), was included in each assay as a control. NTV vaccine in vitro potency values were measured at each of the following three stages: Stage 1—freshly thawed NTV bulk stored at −80 °C (frozen bulk); Stage 2—freshly dried films of NTV vaccine samples; and Stage 3—dried films NTV vaccine sample stored over time at various temperatures (stored dried films).

#### 2.2.5. Characterization of Film Properties

After complete drying, film samples were sputter-coated with a 12 nm gold layer using a Cressington 208HR system to ensure conductivity. Scanning electron microscopy (SEM) analysis was performed using a ZEISS Sigma 360 field-emission microscope (Oberkochen, Germany). Film samples were prepared with marked test surfaces as specified. All samples were imaged in secondary electron mode under high vacuum, with the acceleration voltage optimized to prevent charging effects on hygroscopic surfaces. Fourier transform infrared (FTIR) spectroscopy was conducted on films prepared with and without virus using a Nicolet iS20 spectrometer (Thermo Fisher Scientific, Waltham, MA, USA). Films were analyzed via the attenuated total reflectance (ATR) method with a diamond/ZnSe prism. Spectra were recorded in the range of 4000 to 800 cm^−1^.

#### 2.2.6. Preparation of Dissolving Microneedle Vaccine for Monkeypox Prevention

The optimized thermostable formulation (F2) was used to fabricate DMNs via the micromolding method. Briefly, viral solution was mixed with the optimized excipient stock solution at a fixed 1:1 volume ratio, and the mixture was cast into polydimethylsiloxane (PDMS) microneedle molds (needle height: 860 µm, base height: 400 µm, array of 302 needles in a circular arrangement; Slin/Shengling Medical, Hebei Saisqi Biotechnology Co., Ltd., Hebei, China). The mold cavities were filled by sequential vacuum degassing using a DZF-6020 vacuum oven (Bluepard, Shanghai, China) and centrifugation using a 3-18KS centrifuge (Sigma, Osterode am Harz, Germany). Subsequently, the molds were dried under controlled conditions (25 °C, 20% RH) for 24 h to form solid DMN arrays. The resulting DMN patches were carefully demolded and stored in a desiccator until use. To assess DMN quality, their morphology and integrity were examined using a stereomicroscope (Stereozoom S9i, Leica, Wetzlar, Germany). Dissolution behavior was evaluated by applying a DMN patch to dorsal porcine skin under gentle, sustained thumb pressure for 5 min. After removal, needle tip dissolution was monitored at specified time points under the stereomicroscope.

#### 2.2.7. Immunization and Sample Collection

Specific-pathogen-free (SPF) female *BALB/c* mice (6–8 weeks old) were obtained from SIPEIFU (Beijing Sibeifu Biotechnology Co., Ltd., Beijing, China). Mice were housed in a temperature-controlled environment (22 ± 2 °C) with a 12 h light/dark cycle and free access to sterile food and water. After 1 week of adaptive feeding, mice were randomly divided into 3 groups (n = 5 per group): blank control group, intramuscular (IM) immunization group, and DMN immunization group. Mice in the blank control group received an intramuscular injection of 0.1 mL sterile 10 mM PBS (pH 7.2) into the hind leg muscle; the IM immunization group was administered 0.1 mL of MVA-BN stock solution (5 × 10^8^ CCID_50_/mL) via the same intramuscular injection route. For DMN immunization, the dorsal hair of mice was removed with depilatory cream, and the skin was cleaned with sterile PBS and dried naturally. Each DMN patch (containing ~1 × 10^7^ CCID_50_ of MVA-BN, verified by CCID_50_ assay) was applied to the hairless dorsal skin with a constant pressure for 5 min to ensure complete dissolution of the needles. Prime-boost immunization was conducted on day 0 and day 28 for all groups. Blood samples were collected from the orbital venous plexus at 7, 14 and 28 days post the boost immunization. For the above mouse immunization and blood collection, mice were anesthetized with 2% isoflurane. At the end of the experiments, mice were euthanized using 2% isoflurane (Reward Life Technology, Shenzhen, China), followed by cervical dislocation.

#### 2.2.8. Immunogenicity Detection

MPXV-specific IgG titers were determined using a single Mouse Anti-MPV IgG ELISA Kit (Fine Test, Wuhan, China), which contains all matched monoclonal antibodies and pre-coated reaction components for four MPXV target antigens, including A35R, B6R (extracellular virion proteins), H3L and M1R (mature virion proteins). Serially diluted serum samples (10^2^ to 10^6^ folds) were tested following the kit’s standard protocol. The absorbance at 450 nm (A450) was read by a full-automatic microplate reader. A sample with an A450 value 2.1-fold higher than that of the blank control group was defined as positive, and the antibody titer was expressed as the reciprocal of the maximum positive serum dilution.

The neutralizing antibody titers in serum samples were determined by a 50% plaque reduction neutralization test (PRNT_50_). Briefly, Vero E6 cells were seeded in 12-well plates and incubated overnight at 37 °C, 5% CO_2_ until the cells reached 90–100% confluency. On the day of the assay, serum samples were heat-inactivated at 56 °C for 30 min. An initial 1:20 dilution was prepared, followed by a series of six 4-fold serial dilutions in DMEM supplemented with 2% FBS. The working MPXV stock was diluted to 600 PFU/mL. An equal volume (0.3 mL) of the diluted virus was added to each serum dilution, mixed, and incubated for 1 h at 37 °C for neutralization. After incubation, the culture medium was removed from the cell monolayers, and 0.5 mL of each serum–virus mixture was added to the wells for a 1 h adsorption period at 37 °C. The inoculum was then aspirated, and each well was overlaid with 1 mL of maintenance medium containing 0.9% methylcellulose. Plates were incubated at 37 °C for 5 days to allow plaque development. Subsequently, cells were fixed with 4% neutral-buffered formaldehyde and stained with 0.5% crystal violet solution for plaque enumeration. The neutralization endpoint titer (PRNT_50_) was calculated as the reciprocal serum dilution yielding a 50% reduction in plaques compared to the virus-only control, using point-to-point linear regression. Samples showing less than 50% virus inhibition at the lowest dilution (1:20) were assigned a titer of <20.

#### 2.2.9. Statistical Analysis

Data are expressed as the mean ± standard deviation (SD) of three independent biological replicates. Statistical analysis was performed using GraphPad Prism (version 10.1.2). Specific tests were applied as follows: linear regression for correlation analysis; one-way or two-way analysis of variance (ANOVA) followed by an appropriate post hoc test for comparisons among multiple groups; and corresponding non-parametric tests (e.g., Kruskal–Wallis test) when the assumptions of parametric tests were not met. A *p*-value of less than 0.05 was considered statistically significant.

## 3. Results

### 3.1. Optimization of Buffer Conditions and Establishment of a High-Throughput Infectivity Assay for NTV Formulation Screening

**Figure 1:**
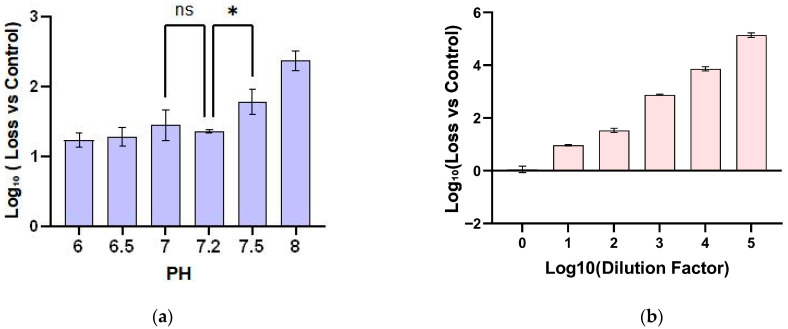
Optimization of buffer conditions and establishment of the RT-qPCR infectivity assay for NTV. (**a**) Effect of pH on NTV vaccine titer after drying, expressed as log_10_ loss relative to the positive control. NTV solutions were dried into films at pH 6.0–8.0 in PBS buffer, data are presented as mean ± SD (n = 3). (**b**) Linear relationship between the relative expression of NTV *E9L* gene and the initial inoculation titer (Log_10_ CCID_50_) at 24 h post-infection, data are presented as mean ± SD (n = 3). Statistical analysis: ANOVA, ns: non-significant, *: *p* < 0.05.

PH is a key factor affecting the stability of live virus vaccines during drying and storage [27]. To optimize the buffer system for the NTV vaccine film formulation, we evaluated the effect of pH on NTV infectivity after drying. Phosphate-buffered saline (PBS) was selected as the buffer system for its effective buffering capacity within the pH range of 6.0–8.0 and physiological ionic strength compatibility. NTV vaccine solutions were adjusted to target pH values (6.0–8.0), mixed with hyaluronic acid (HA) solution, then dried into films at 4 °C for 24 h, with freshly thawed virus solution serving as the positive control. Comparative analysis showed that all dried samples exhibited a significant reduction in viral titer compared with the positive control, indicating notable viral activity degradation during the drying process. However, no statistically significant difference in NTV stability was observed when drying was performed at pH 6.0–7.5 (ANOVA, *p* = 0.345). In contrast, drying at pH 8.0 resulted in an approximate 1 log_10_ reduction in viral titer (Figure 1a), which may be attributed to the combined stress of alkaline environment and drying process. Based on these results, 10 mM PBS at pH 7.2 was selected as the buffer system for all subsequent experiments, as it showed optimal drying performance and is the standard buffer used in clinical-stage NTV vaccine products.

While the CCID_50_ assay is the gold standard for assessing poxvirus infectivity, its time-consuming nature and low throughput cannot meet the demands of large-scale excipient screening. To address this limitation, we established an RT-qPCR-based surrogate method for high-throughput quantification of NTV infectivity. After determining the NTV titer via the standard CCID_50_ assay, gradient-diluted NTV solutions were used to infect BHK-21 cell monolayers, and the relative mRNA expression level of the *E9L* gene was detected by RT-qPCR, with *β-actin* as the endogenous reference gene (CV < 5% for Ct values). Linear regression analysis revealed a highly significant positive correlation between the relative expression of *E9L* mRNA (2^−ΔΔCt^) and the initial inoculation titer (Log_10_ CCID_50_), with the fitted equation Y = 0.9939X − 0.08853 (R^2^ = 0.9867, *p* < 0.0001) and a 95% confidence interval for the slope of 0.9367 to 1.051 (Figure 1b). This assay had a practical detection limit of at least 10^3^ CCID_50_/mL, showing high sensitivity and reliability.

### 3.2. Establishment of Screening System and Primary Screening of Matrix Excipients for NTV Stabilization

**Figure 2:**
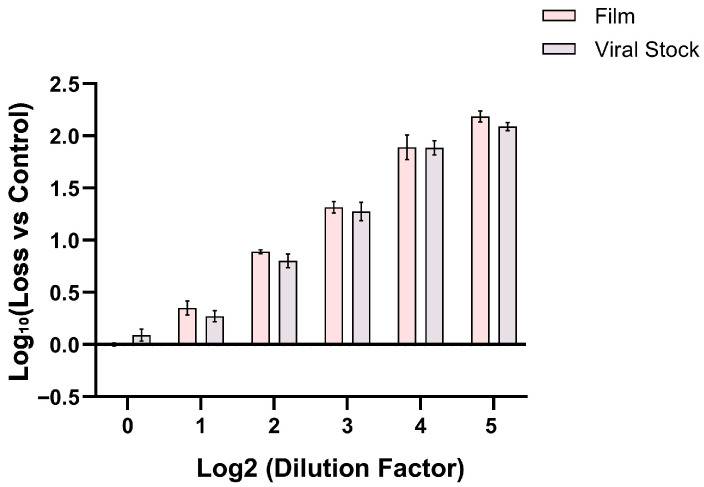
Comparison of Log_10_ Values of Viral Particle Loss Between Material Films and Viral Stock at Different Dilution Factors. This figure presents the Log_10_ values of viral particle loss relative to the control group for dried material films loaded with NTV (Film) and viral stock (Viral Stock) under different dilution factors. All samples contained 15% PVA (n = 3). Two-way ANOVA showed that the DNA content of samples did not differ significantly between different treatment groups (*p* > 0.05), indicating that the decrease in infectious viral titer in dried material films is not attributed to the physical loss of viral particles.

After drying and storage with common stabilizers in PBS (4 °C, 2 weeks), the NTV showed a titer reduction exceeding 1 log_10_. These storage conditions provided a good “stability window” to screen for stabilizers of the NTV in the dried state (Figure 1a). No significant difference was observed in the loss of serially diluted virus particles incorporated within the material film after drying compared to frozen controls (Figure 2). This indicates that the reduction in NTV infectious viral titer was not attributable to physical loss of viral particles. Therefore, the ability of various additives to improve NTV titer retention can be demonstrated beyond assay variability. The ability of various classes and types of pharmaceutical excipients to stabilize NTV in a vaccine was determined using the experimental shown in methods. Briefly, NTV bulk virus was mixed with various excipients, dried in microcentrifuge tubes, stored at 4 °C and 25 °C for two weeks, rehydrated, and then, examined its infectivity by RT-qPCR.

Different excipient categories and types could stabilize NTV by different mechanisms upon drying in MAPs and/or during storage in the solid state [28]. To prioritize stabilizers with direct clinical translation potential, we selectively evaluated 16 candidate excipients (including molecular weight variations, Table 1), all of which are commonly employed in DMN and are also found on the FDA inactive ingredient guide for parenteral products (with a few exceptions), were evaluated for their effect on NTV stability. Commonly employed formulating excipients include dextran, hydroxyethyl starch [15], carboxymethyl cellulose (CMC) [29], polyvinylpyrrolidone (PVP) [30], polyvinyl alcohol (PVA) [31], and hyaluronic acid (HA) [32], among others. Stabilizers are typically sugars, such as trehalose [32], maltose [33], sucrose [34], and xylitol [29]. The excipient concentrations evaluated were selected to ensure efficient evaporative drying in the centrifuge tubes.

**Figure 3:**
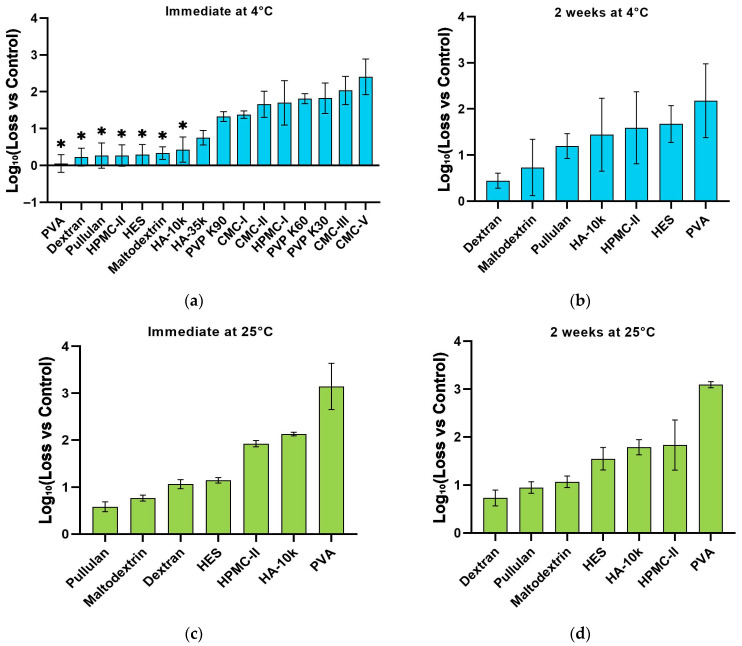
Evaluation of candidate excipients on the stability of NTV viral infectious titer during drying and storage. All film samples were prepared by mixing equal volumes of viral solution and material solution, resulting in a total volume of 150 µL. (**a**,**b**) Viral titer loss after drying and two-week storage at 4 °C. Seven excipients that demonstrated minimal loss (hits) were selected for further analysis. The asterisk (*) in panel (**a**) indicates these seven hit candidates. (**c**,**d**) Stability assessment of the seven hit excipients at 25 °C. (**c**) Titer loss immediately post-drying at 25 °C. (**d**) Titer loss after two-week storage at 25 °C following the drying process. Data are presented as mean ± SD. The *y*-axis represents the logarithm of the infectious titer loss relative to the control.

Seven excipient candidates demonstrating minimal loss of NTV infectious titer after 4 °C drying and storage were designated as ‘hits’ (Figure 3a,b). Subsequently, these seven hits underwent further stability assessment under accelerated storage conditions at 25 °C (Figure 3c,d). Following two weeks of storage and drying at this temperature, pullulan and dextran emerged as the most effective stabilizers, exhibiting a remarkably low infectious titer reduction of less than 1 log_10_. While pullulan demonstrated optimal stabilization characteristics, its lack of regulatory approval for human administration precluded its selection [35]. Therefore, dextran—exhibiting comparable stabilization efficacy—was selected. As a clinically established plasma expander, dextran possesses an extensive safety profile with no reported cytotoxicity. Critically, approved clinical protocols already employ dextran within soluble microneedle systems for transdermal drug delivery, thereby supporting its suitability as the stabilizer [36].

### 3.3. Screening of Synergistic Stabilizers and Determination of the Optimal Formulation

**Figure 4:**
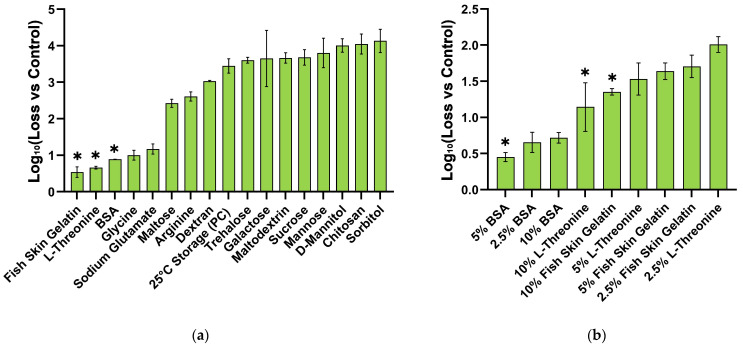
Screening and optimization of supplementary stabilizers in combination with the primary excipient, dextran. (**a**) Effect of individual supplementary stabilizers (all formulated with dextran) on NTV viral infectious titer after drying and two-week storage at 25 °C. (**b**) Titration of promising stabilizers to determine their optimal concentrations for stabilizing NTV when combined with dextran, after drying and two-week storage at 25 °C. Data are presented as mean ± SD (n = 3). Asterisk (*) indicates candidate stabilizers and their concentrations. Note: The abbreviation “PC” denotes the positive control sample stored at 25 °C.

Following the identification of the primary excipient, we screened supplementary stabilizers to further enhance viral stability within the formulation. We employed the same film-forming method to incorporate various supplementary stabilizers into the formulation (virus combined with the primary excipient), maintaining a constant pH. The infectious titer of the mixture was then monitored over a two-week period following drying and storage at 25 °C to assess stability under these conditions. Finally, we identified three distinct stabilizer that maintained comparable or elevated infectious titers relative to the no-stabilizer control upon drying and storage. These included hydrolyzed gelatin, BSA and L-threonine (Figure 4a).

To further evaluate these stabilizer combinations and to determine their optimal effective concentrations, each stabilizer was titrated, from 0.5- to 1.5-fold range of concentration used in the initial screen, for their stabilizing effect on NTV during drying and storage at 25 °C, 2 weeks. As shown in Figure 4b, 10% L-Threonine, 10% Fish Skin Gelatin and 5% BSA looked promising, with the latter two as common stabilizers for NTV.

**Figure 5:**
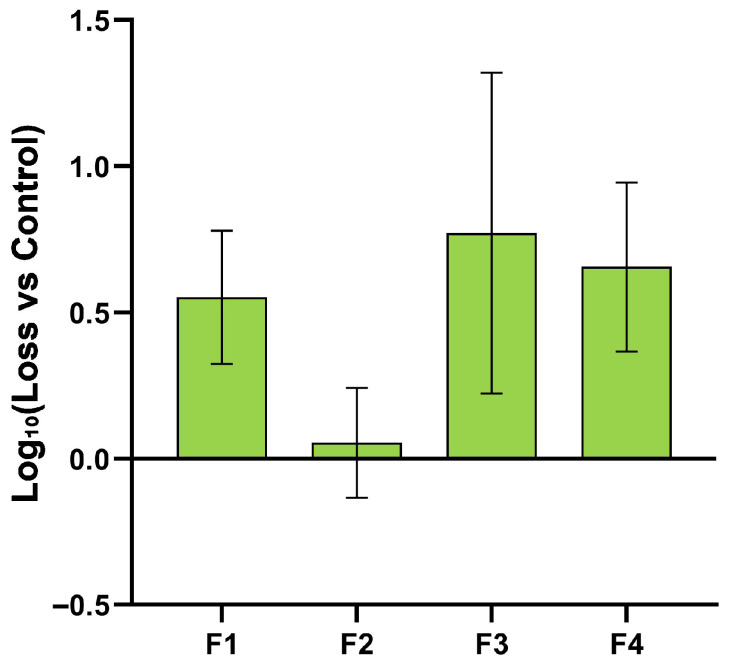
Screening and evaluation of combinations of the top-performing stabilizers. Stability of NTV infectious titer for each combination formulation after drying at 25 °C. The combination formulations, particularly F2, demonstrated enhanced stability with minimal titer loss compared to individual stabilizers. Data are presented as mean ± SD (n = 3). The vertical axis represents the logarithmic value of infectious titer loss relative to the frozen control.

To investigate potential synergistic or enhancing effects among the top-performing stabilizers, we screened combinations incorporating three top individual stabilizers (formulations F1–F4, Table 2). As depicted in Figure 5, the dried combination formulations exhibited a trend of enhanced stability, yielding lower infectivity losses. Formulation F2 was identified as the optimal choice. However, due to the potential immunogenicity of its component, BSA, human serum albumin (HSA) may be considered as a substitute protective agent in subsequent preclinical studies.

### 3.4. Accelerated and Real-Time Stability Studies with Candidate NTV Formulations in the Dried State

**Figure 6:**
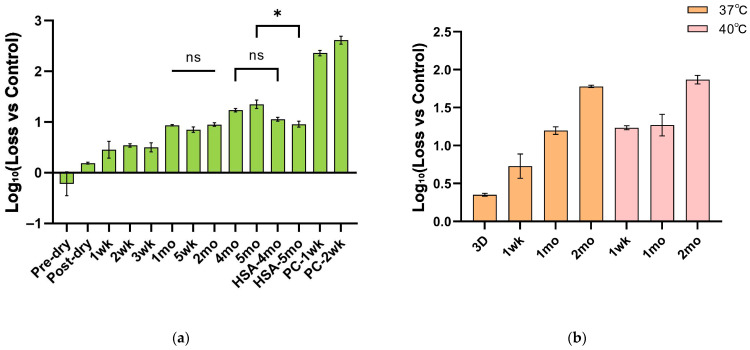
Thermal stability evaluation of the optimized formulation. (**a**) Stability data of the optimized formulation during long-term storage at 25 °C, expressed as mean ± standard deviation. (**b**) Accelerated stability of the formulation at 37 °C and 40 °C. Data are expressed as mean ± standard deviation (n = 3). ns, non-significant; *, *p* < 0.05.

We first evaluated the long-term stability of the candidate NTV formulation under ambient conditions (25 °C). It exhibited a titer loss of only approximately 1 log_10_ over a two-month storage period. The optimized formulation, where BSA was replaced with HSA, demonstrated superior performance over a four-month storage period, with the loss maintained at around 1 log_10_ (Figure 6a). Due to unequal variances, a non-parametric test was applied. The results showed that after 4 months of storage, there was no significant difference in viral activity between the BSA and HSA-containing formulations (ANOVA, *p* = 0.9047). However, after 5 months, the HSA-based formulation provided superior stabilization compared to its BSA counterpart, showing a statistically significant advantage (ANOVA, *p* = 0.0107). These data demonstrate that HSA is functionally equivalent and represents a viable, potentially superior, alternative stabilizer. This stands in stark contrast to the control group stored at 25 °C (containing only the viral solution), which lost more than 2 log_10_ in potency after just one week of storage at 25 °C, highlighting the significant advantage of the formulation in preserving NTV infectivity. Furthermore, it is noteworthy that the viral titer loss approached a plateau approximately one month after drying, with no statistically significant further decline observed between months 1 and 2 (ANOVA, *p* = 0.0679). This phenomenon is likely attributable to the moisture content within the dried film reaching an equilibrium and stabilizing beyond this time point. Concurrently, the formulation also exhibited robust stability under the more stringent condition of 37 °C and 40 °C (Figure 6b).

### 3.5. Physical Characterization of Thin Films

**Figure 7:**
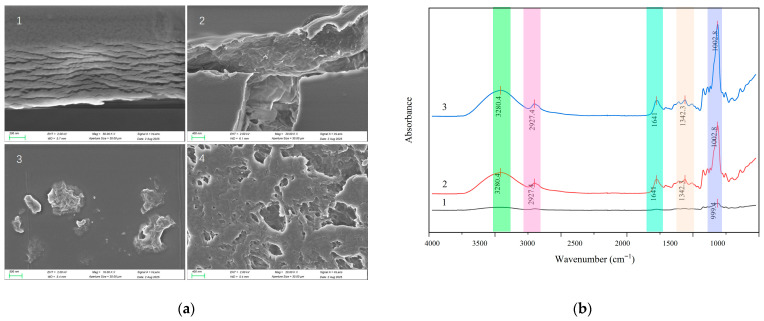
Physical characterization of the optimized thin-film formulation. (**a**) SEM images illustrating the structural evolution of the films: (1) Surface morphology of the film prepared with base excipients. (2) The optimized virus-free formulation exhibits a glassy-smooth surface and a densely packed internal structure. (3) Virus-loaded film showing distinct surface protrusions. (4) Cross-section of the virus-loaded film revealing a transformation into a hierarchically porous, sponge-like network. (**b**) FTIR spectroscopic analysis of (1) the base film, (2) the optimized film containing stabilizers, and (3) the final film loaded with the NTV.

SEM characterization revealed that thin films prepared with base excipients exhibited smooth surfaces and laminated cross-sectional morphology, while optimized virus-free formulations displayed glassy-smooth surfaces with densely packed internal structures. Upon incorporating viruses into the formulation followed by drying, virus-loaded films developed distinct surface protrusions and transformed into hierarchically porous sponge-like networks in cross-section (Figure 7a). This structural reorganization may synergistically contribute to stabilization via two potential pathways: the porous architecture physically isolates viral particles while buffering mechanical stress; and closed-pore structures form oxygen barriers with molecular-level viral embedding fixing viral conformation [37].

FTIR spectroscopy was used to identify bond interactions between each component of the optimized film and the NTV. The results demonstrated that incorporating stabilizers and NTV profoundly altered the hydrogen-bonding network in the film matrix. At the critical wavenumber of 3280 cm^−1^ (N-H/O-H stretching vibration), absorbance values showed a substantial stepwise increase across three sample groups (0.03 → 0.20 → 0.24). Significantly, absorbance at 999.91 cm^−1^ (C-O-C glycosidic bond) surged from 0.64 to 0.86 upon viral incorporation, suggesting the potential formation of specific interactions between viral glycoproteins and the dextran backbone. Concurrently, absorbance at 601 cm^−1^ increased from 0.34 to 0.40, indicating virus-induced enhancement of the film’s structural rigidity (Figure 7b). It is important to note that the FTIR data reflect trends in overall absorbance; these changes are consistent with the formation of hydrogen bonds and glycosidic bonds, but this study did not directly identify the precise interactions between specific viral proteins and film components. Taken together, the data support the hypothesis that NTV stabilization may rely on a dual mechanism: hydrogen bonding via N-H/O-H groups with protectants and potential interactions between viral surface glycoproteins and the polysaccharide matrix [37].

### 3.6. Morphology Characterization and In Vitro Dissolution Performance of Optimized Dissolving Microneedles

**Figure 8:**
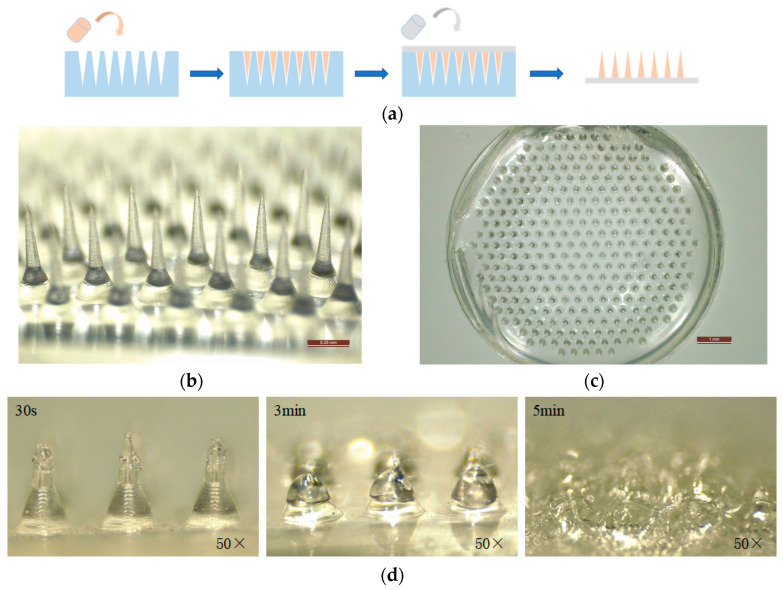
Preparation process and morphological characterization of the optimized DMN patches. (**a**) Schematic flow diagram of the DMN patch preparation process, including formulation casting, vacuum degassing and centrifugation filling, controlled drying, and demolding. (**b**) High-magnification stereomicroscopic image of a single microneedle, showing intact tip morphology and conical structure consistent with the mold design. (**c**) Top-view stereomicroscopic image of the overall microneedle array, with a uniform and complete structure without breakage or cavities. (**d**) In vitro dissolution process of the optimized DMN patches on excised porcine skin.

We verified the feasibility of translating the optimized thermostable film formulation (F2) into DMN patches (Figure 8a). The prepared DMNs exhibited a well-defined conical structure with intact sharp tips, which was fully consistent with the design of the custom PDMS molds (Figure 8b). The overall microneedle array was uniform and complete, with no observable tip breakage, cavities or structural defects (Figure 8c). In vitro dissolution test on excised porcine skin showed that the DMNs initiated dissolution within 30 s after skin insertion, the main needle body was almost completely dissolved at 3 min, and full dissolution of the needle base was achieved within 5 min (Figure 8d). The virus titer in a single DMN patch was determined by CCID_50_ assay to be approximately 1 × 10^7^ CCID_50_. These results confirmed that the optimized formulation can be reliably fabricated into qualified DMN patches with rapid dissolution properties, supporting subsequent in vivo vaccination experiments.

### 3.7. Immunogenicity of MVA-BN-Loaded Dissolving Microneedle Patches in Mice

**Figure 9:**
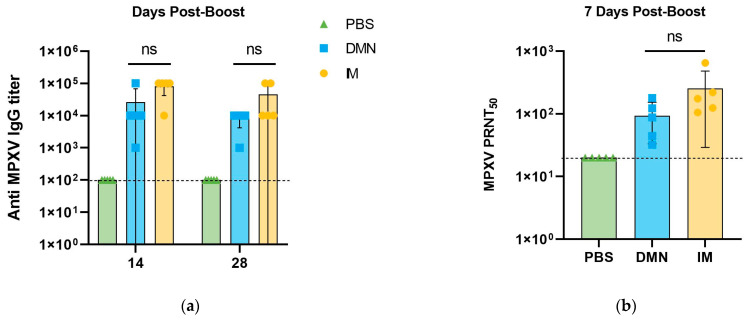
Immunogenicity of MVA-BN-loaded DMN patches in mice. (**a**) Log_10_-transformed endpoint titers of MPXV-specific total IgG in mouse serum at 14 and 28 days post-boost immunization, detected by a commercial combined ELISA kit pre-coated with four MPXV antigens in a single well. (**b**) PRNT_50_ endpoint titers of MPXV neutralizing antibodies in mouse serum at 7 days post-boost immunization. The dashed line in both panels indicates the mean value of the PBS blank control group, defined as the lower detection limit of the corresponding assay. Data are presented as mean ± SD (n = 5); ns, non-significant (*p* > 0.05).

The core objective of this study was to develop a thermostable dissolvable microneedle (DMN) formulation platform suitable for replication-deficient orthopoxvirus vaccines for monkeypox prevention. For this purpose, we used the clinical-stage NTV strain for systematic formulation optimization and in vitro thermostability evaluation. Given the highly conserved immunodominant antigen profile and the ability to induce cross-protective immunity against MPXV between the two replication-deficient orthopoxviruses, we selected the globally licensed MVA-BN strain as the validation strain to assess the in vivo immunogenicity of our formulation platform. Based on the optimized thermostable formulation screened with NTV, we prepared MVA-BN-loaded DMN patches, and further evaluated their in vivo immunogenicity via a prime-boost immunization regimen in *BALB/c* mice. MPXV-specific IgG endpoint titers in serum were detected at 14 and 28 days post-boost immunization, and MPXV neutralizing antibody titers were determined at 7 days post-boost immunization. As shown in Figure 9a, both the intramuscular (IM) immunization group and DMN immunization group induced high levels of MPXV-specific IgG antibodies against the four pre-coated immunodominant MPXV antigens at 14 and 28 days post-boost, with log_10_-transformed endpoint titers reaching approximately 4.0 for most mice in both groups. No statistically significant difference was observed in serum IgG endpoint titers between the IM and DMN groups at both time points (ANOVA, *p* > 0.05). The dashed line in the figure indicates the mean value of the PBS group, which was defined as the lower detection limit of the assay. The neutralizing activity against MPXV was assessed by PRNT_50_ assay (Figure 9b). Both the IM and DMN groups showed detectable MPXV-specific neutralizing antibodies at 7 days post-boost immunization, which was attributed to the cross-protective immune response induced by the MVA-BN vaccine. There was no statistically significant difference in PRNT_50_ endpoint titers between the two immunization groups (ANOVA, *p* > 0.05). These results confirmed that the DMN patch prepared with the optimized thermostable formulation can induce effective humoral immune responses in vivo, with immunogenicity non-inferior to the traditional intramuscular injection route.

## 4. Discussion

While microarray patch (MAP) delivery has achieved remarkable progress in the stabilization of inactivated and replicating live attenuated vaccines [21,24], translating these technologies to replication-deficient orthopoxvirus vaccines remains a major challenge. These genetically attenuated viruses with abrogated replication capacity lack self-repair mechanisms, rendering them far more vulnerable to dehydration- and storage-induced infectivity loss than other vaccine types [38,39,40]. Additionally, their stability evaluation depends on complex cell-based infectivity assays rather than simple antigen quantification, which creates an additional barrier to high-throughput formulation development for DMN delivery. Given these unique challenges, establishing a reliable formulation development workflow for replication-deficient NTV requires addressing both the viral instability during processing and the low-throughput limitation of traditional infectivity assays. Traditional CCID_50_ assays, the gold standard for potency assessment, are time-consuming and impractical for large-scale excipient screening. To this end, we first established and validated an RT-qPCR-based surrogate assay targeting the early viral *E9L* transcript, which showed a strong linear correlation (R^2^ > 0.98) with the gold-standard CCID_50_ assay. This platform enabled high-throughput, objective and sensitive infectivity quantification, with a detection limit of 10^3^ CCID_50_/mL, making it ideal for rapid formulation screening.

Using this validated assay, we developed a film-based screening model simulating the microneedle drying process to streamline DMN formulation development. Through this integrated workflow, we systematically screened 16 clinical-grade excipients, and identified the optimal formulation F2 with dextran as the core matrix, combined with L-threonine and BSA as synergistic stabilizers. The final optimized formulation F2 effectively minimized NTV potency loss during drying and long-term ambient storage, with only ~1 log_10_ titer loss after 2 months of storage at 25 °C and <1 log_10_ loss after 1 week at 37 °C. Leveraging the SEM and FTIR characterization results, we preliminarily explored the potential stabilization mechanism underlying the excellent performance of the optimized formulation. As the core matrix, dextran with abundant hydroxyl groups is hypothesized to form an extensive hydrogen-bonding network, which may restrict viral particle motion and reduce protein denaturation during drying and storage [41]. L-threonine may mimic the hydrated state of viral proteins via hydrogen bonding to alleviate dehydration-induced damage, while BSA may mitigate oxidative damage and maintain viral protein conformation, with the two potentially acting synergistically to preserve viral infectivity [42,43,44]. We further confirmed that HSA can serve as a clinically compliant alternative to BSA with comparable stabilization efficacy, supporting the clinical translation potential of this formulation. Although pullulan exhibited better stabilization performance in screening, it was not selected due to limited parenteral regulatory approval, and its application potential will be further explored in subsequent work.

Building on the excellent thermostability of the film formulation, we successfully translated it into functional DMN patches via a simple micromolding method. The fabricated DMNs exhibited intact conical structure with sharp tips, and achieved complete dissolution in porcine skin within 5 min, meeting the requirements for rapid intradermal vaccine delivery. We further verified the in vivo application potential of this formulation platform in a mouse model, using the globally licensed MVA-BN strain as a surrogate for NTV. This strain selection strategy is scientifically justified by the high conservation of immunodominant antigens between the two replication-deficient orthopoxviruses, which can induce cross-protective immune responses against MPXV [15]. The in vivo results showed that freshly prepared MVA-BN-loaded DMNs induced robust MPXV-specific IgG antibodies and detectable cross-neutralizing antibodies in mice, with no statistically significant difference in immunogenicity compared to the traditional intramuscular injection route. These findings confirm that the optimized thermostable formulation not only preserves viral infectivity in vitro, but also maintains the immunogenicity of the vaccine in vivo, realizing the core goal of developing a DMN-based thermostable orthopoxvirus vaccine for monkeypox prevention.

Nevertheless, this study has two main limitations that need to be addressed in subsequent research. First, the thermal stability assessment of the formulation in this study is mainly based on RT-qPCR data, and further systematic evaluation of the long-term thermostability of the formulation using the gold-standard CCID_50_ infectivity assay is required to directly confirm the retention of viral infectious titer during ambient storage. Second, this study only verified the immunogenicity of freshly prepared DMN patches, and the in vivo immunogenicity of the vaccine after long-term ambient temperature storage remains to be further validated, which is the core evidence for the cold chain-free application of this vaccine platform. We will carry out the above two works in our follow-up research to further improve the scientific integrity and clinical translation potential of this study.

## 5. Conclusions

This study completed the full workflow of formulation screening, dissolvable microneedle (DMN) fabrication and in vivo immunogenicity evaluation of a replication-deficient orthopoxvirus vaccine for monkeypox prevention. We successfully developed a thermostable NTV film formulation with excellent ambient temperature storage stability, which can be reliably fabricated into rapidly dissolving DMN patches. Using the globally licensed MVA-BN strain as a surrogate, we verified that the optimized DMN patches induced robust MPXV-specific IgG and neutralizing antibodies in mice, with immunogenicity non-inferior to traditional intramuscular injection. This strategy offers a potential approach to alleviate the core limitations of cold-chain dependence and inconvenient administration of current orthopoxvirus vaccines, provides supporting data for the development of ambient temperature-storable Mpox vaccines, and may unlock broad potential for the further development of diverse dissolvable microneedle-vectored vaccines by leveraging NTV and MVA-BN as mature replication-deficient viral vector platforms [45].

## 6. Patents

The invention related to the work reported in this manuscript has filed a patent application (A Soluble Microneedle Patch for Mpox Vaccine and Its Preparation Method, Application No. 202610106175.3) with the China National Intellectual Property Administration, which was accepted on 27 January 2026.

## Figures and Tables

**Table 1:** Summary of excipients screened for film formulation development.

Name	Category/ Type	Specification	Tested Concentration (*w*/*v*)	Name	Category/ Type	Specification	Tested Concentration (*w*/*v*)
PVA	Polymer	9–10 kDa	30%	HA-35K	Glycosaminoglycan	30–45 kDa	30%
HPMC-I	Polymer	4000 mPa.s	4%	Dextran	Polysaccharide	70 kDa	40%
HPMC-II	Polymer	4000–6500 mPa.s	4%	Pullulan	Polysaccharide	Purity 99%	30%
PVP K30	Polymer	58 kDa	40%	HES	Polysaccharide	110–150 kDa	40%
PVP K60	Polymer	220 kDa	40%	CMC-I	Cellulose Derivative	DS = 1.2	4%
PVP K90	Polymer	~1300 kDa	40%	CMC-II	Cellulose Derivative	DS = 1.2	4%
Maltodextrin	Oligosaccharide	Variable (DE 10–20)	60%	CMC-III	Cellulose Derivative	DS = 0.9	4%
HA-10K	Glycosaminoglycan	10 kDa	40%	CMC-V	Cellulose Derivative	DS = 0.7	4%

**Table 2:** Design of four candidate formulation combinations (F1–F4) based on the three top-performing individual stabilizers in combination with the primary dextran-based formulation.

Formulation	5% BSA	10% Fish Skin Gelation	10% L-Threonine	Dextran
F1	√			√
F2	√		√	√
F3	√	√		√
F4		√	√	√

The checkmark (√) denotes that the component is included in the formulation.

## Data Availability

Data will be made available on request.

## Abbreviations

The following abbreviations are used in this manuscript:

ANOVA	Analysis of Variance
ATR	Attenuated total reflectance
BIBP	Beijing Institute of Biological Products
BSA	Bovine Serum Albumin
CMC	Carboxymethyl cellulose
CPE	Cytopathic effect
CCID_50_	50% Cell Culture Infective Dose
DMEM	Dulbecco’s Modified Eagle Medium
DMN	Dissolving Microneedle
EDTA	Ethylenediaminetetraacetic acid
FDA	Food and Drug Administration
FBS	Fetal bovine serum
FTIR	Fourier Transform Infrared
HA	Hyaluronic acid
HPMC	Hydroxypropyl methylcellulose
HSA	Human Serum Albumin
LC16m8	Lister strain Clone, m8 passage
LMICs	Low- and middle-income countries
MAP	Microneedle Array Patch
MeRu	Measles–Rubella
MEM	Minimum Essential Medium
MPOX	Monkeypox
MPXV	Monkeypox Virus
MVA-BN	Modified Vaccinia Ankara-Bavarian Nordic
NTV	Non-replicating Tiantan Vaccinia Virus
PBS	Phosphate-buffered saline
PC	Positive Control
PDMS	Polydimethylsiloxane
PVA	Polyvinyl alcohol
PVP	Polyvinylpyrrolidone
QC	Quality Control
SD	Standard Deviation
SEM	Scanning Electron Microscopy
tIPV	Trivalent Inactivated Polio Vaccine
WHO	World Health Organization

## References

[1] Peng Q., Xie Y., Kuai L., Wang H., Qi J., Gao G.F., Shi Y. (2023). Structure of monkeypox virus DNA polymerase holoenzyme. Science.

[2] Datler J., Hansen J.M., Thader A., Schlogl A., Bauer L.W., Hodirnau V.V., Schur F.K.M. (2024). Multi-modal cryo-EM reveals trimers of protein A10 to form the palisade layer in poxvirus cores. Nat. Struct. Mol. Biol..

[3] Rao A.K., Schulte J., Chen T.H., Hughes C.M., Davidson W., Neff J.M., Markarian M., Delea K.C., Wada S., Liddell A. (2022). Monkeypox in a Traveler Returning from Nigeria—Dallas, Texas, July 2021. MMWR Morb. Mortal. Wkly. Rep..

[4] Ferrareze P.A.G., Pereira E.C.R.A., Thompson C.E. (2023). Genomic characterization and molecular evolution of human monkeypox viruses. Arch. Virol..

[5] Prasad S., Galvan Casas C., Strahan A.G., Fuller L.C., Peebles K., Carugno A., Leslie K.S., Harp J.L., Pumnea T., McMahon D.E. (2023). A dermatologic assessment of 101 mpox (monkeypox) cases from 13 countries during the 2022 outbreak: Skin lesion morphology, clinical course, and scarring. J. Am. Acad. Dermatol..

[6] O’Toole A., Neher R.A., Ndodo N., Borges V., Gannon B., Gomes J.P., Groves N., King D.J., Maloney D., Lemey P. (2023). APOBEC3 deaminase editing in mpox virus as evidence for sustained human transmission since at least 2016. Science.

[7] Schultz-Pernice I., Fahmi A., Brito F., Liniger M., Chiu Y.C., David T., Oliveira Esteves B.I., Golomingi A., Zumkehr B., Gerber M. (2025). Monkeypox virus spreads from cell-to-cell and leads to neuronal death in human neural organoids. Nat. Commun..

[8] Tamir H., Noy-Porat T., Melamed S., Cherry-Mimran L., Barlev-Gross M., Alcalay R., Yahalom-Ronen Y., Achdout H., Politi B., Erez N. (2024). Synergistic effect of two human-like monoclonal antibodies confers protection against orthopoxvirus infection. Nat. Commun..

[9] Bertran M., Andrews N., Davison C., Dugbazah B., Boateng J., Lunt R., Hardstaff J., Green M., Blomquist P., Turner C. (2023). Effectiveness of one dose of MVA-BN smallpox vaccine against mpox in England using the case-coverage method: An observational study. Lancet Infect. Dis..

[10] Wang Y., Yang K., Zhou H. (2023). Immunogenic proteins and potential delivery platforms for mpox virus vaccine development: A rapid review. Int. J. Biol. Macromol..

[11] Garcia-Atutxa I., Mondragon-Teran P., Huerta-Saquero A., Villanueva-Flores F. (2024). Advancements in monkeypox vaccines development: A critical review of emerging technologies. Front. Immunol..

[12] Drennan P.G., Provine N.M., Harris S.A., Otter A., Hollett K., Cooper C., De Maeyer R.P.H., Nassanga B., Ateere A., Pudjohartono M.F. (2025). Immunogenicity of MVA-BN vaccine deployed as mpox prophylaxis: A prospective, single-centre, cohort study and analysis of transcriptomic predictors of response. Lancet Microbe.

[13] Srivastava S., Kumar S., Jain S., Mohanty A., Thapa N., Poudel P., Bhusal K., Al-Qaim Z.H., Barboza J.J., Padhi B.K. (2023). The Global Monkeypox (Mpox) Outbreak: A Comprehensive Review. Vaccines.

[14] Kobiyama K., Utsumi D., Kaku Y., Sasaki E., Yasui F., Okamura T., Onodera T., Tobuse A.J., Sakkour A., Amiry A.F. (2025). Immunological analysis of LC16m8 vaccine: Preclinical and early clinical insights into mpox. eBioMedicine.

[15] Chu Q., Huang B., Li M., Cheng X., Huo S., Ren J., Deng Y., Tan W. (2023). Non-replicating vaccinia virus NTV as an effective next-generation smallpox and monkeypox vaccine: Evidence from mouse and rhesus monkey models. Emerg. Microbes Infect..

[16] Iwata H., Kakita K., Imafuku K., Takashima S., Haga N., Yamaguchi Y., Taguchi K., Oyamada T. (2022). Safety and dose-sparing effect of Japanese encephalitis vaccine administered by microneedle patch in uninfected, healthy adults (MNA-J): A randomised, partly blinded, active-controlled, phase 1 trial. Lancet Microbe.

[17] Berger M.N., Mowbray E.S., Farag M.W.A., Mathieu E., Davies C., Thomas C., Booy R., Forster A.H., Skinner S.R. (2023). Immunogenicity, safety, usability and acceptability of microarray patches for vaccination: A systematic review and meta-analysis. BMJ Glob. Health.

[18] Sartawi Z., Blackshields C., Faisal W. (2022). Dissolving microneedles: Applications and growing therapeutic potential. J. Control. Release.

[19] Caudill C., Perry J.L., Iliadis K., Tessema A.T., Lee B.J., Mecham B.S., Tian S., DeSimone J.M. (2021). Transdermal vaccination via 3D-printed microneedles induces potent humoral and cellular immunity. Proc. Natl. Acad. Sci. USA.

[20] Tang Z., Su T., Jiang T., Hu J., Chen D., Li X., Lu J., Lin J., Shen T. (2025). Development of a dissolving microneedle patch for transdermal delivery of SARS-CoV-2 mRNA vaccine with enhanced stability and immunogenicity. J. Control. Release.

[21] Edwards C., Shah S.A., Gebhardt T., Jewell C.M. (2023). Exploiting Unique Features of Microneedles to Modulate Immunity. Adv. Mater..

[22] Adigweme I., Yisa M., Ooko M., Akpalu E., Bruce A., Donkor S., Jarju L.B., Danso B., Mendy A., Jeffries D. (2024). A measles and rubella vaccine microneedle patch in The Gambia: A phase 1/2, double-blind, double-dummy, randomised, active-controlled, age de-escalation trial. Lancet.

[23] Adediran E., Arte T., Pasupuleti D., Vijayanand S., Singh R., Patel P., Gulani M., Ferguson A., Uddin M., Zughaier S.M. (2025). Delivery of PLGA-Loaded Influenza Vaccine Microparticles Using Dissolving Microneedles Induces a Robust Immune Response. Pharmaceutics.

[24] Donnelly R.F., Prausnitz M.R. (2024). The promise of microneedle technologies for drug delivery. Drug Deliv. Transl. Res..

[25] Donadei A., Kraan H., Ophorst O., Flynn O., O’Mahony C., Soema P.C., Moore A.C. (2019). Skin delivery of trivalent Sabin inactivated poliovirus vaccine using dissolvable microneedle patches induces neutralizing antibodies. J. Control. Release.

[26] Wan Y., Gupta V., Bird C., Pullagurla S.R., Fahey P., Forster A., Volkin D.B., Joshi S.B. (2021). Formulation Development and Improved Stability of a Combination Measles and Rubella Live-Viral Vaccine Dried for Use in the Nanopatch(TM) Microneedle Delivery System. Hum. Vaccin. Immunother..

[27] Joyce J.C., Collins M.L., Rota P.A., Prausnitz M.R. (2021). Thermostability of Measles and Rubella Vaccines in a Microneedle Patch. Adv. Ther..

[28] Choo J.J.Y., McMillan C.L.D., Fernando G.J.P., Hall R.A., Young P.R., Hobson-Peters J., Muller D.A. (2021). Developing a Stabilizing Formulation of a Live Chimeric Dengue Virus Vaccine Dry Coated on a High-Density Microarray Patch. Vaccines.

[29] Moon S.S., Richter-Roche M., Resch T.K., Wang Y., Foytich K.R., Wang H., Mainou B.A., Pewin W., Lee J., Henry S. (2022). Microneedle patch as a new platform to effectively deliver inactivated polio vaccine and inactivated rotavirus vaccine. npj Vaccines.

[30] Ray S., Wirth D.M., Ortega-Rivera O.A., Steinmetz N.F., Pokorski J.K. (2022). Dissolving Microneedle Delivery of a Prophylactic HPV Vaccine. Biomacromolecules.

[31] Wang Y., Li S., Dong C., Ma Y., Song Y., Zhu W., Kim J., Deng L., Denning T.L., Kang S.M. (2021). Skin vaccination with dissolvable microneedle patches incorporating influenza neuraminidase and flagellin protein nanoparticles induces broad immune protection against multiple influenza viruses. ACS Appl. Bio Mater..

[32] Bagwe P., Bajaj L., Menon I., Braz Gomes K., Kale A., Patil S., Vijayanand S., Gala R., D’Souza M.J., Zughaier S.M. (2023). Gonococcal microparticle vaccine in dissolving microneedles induced immunity and enhanced bacterial clearance in infected mice. Int. J. Pharm..

[33] Braz Gomes K., D’Souza B., Vijayanand S., Menon I., D’Souza M.J. (2022). A dual-delivery platform for vaccination using antigen-loaded nanoparticles in dissolving microneedles. Int. J. Pharm..

[34] Perez Cuevas M.B., Kodani M., Choi Y., Joyce J., O’Connor S.M., Kamili S., Prausnitz M.R. (2018). Hepatitis B vaccination using a dissolvable microneedle patch is immunogenic in mice and rhesus macaques. Bioeng. Transl. Med..

[35] Singh R.S., Kaur N., Hassan M., Kennedy J.F. (2021). Pullulan in biomedical research and development—A review. Int. J. Biol. Macromol..

[36] He Y., Zhu B., Qiu H., You L., Lin L., Pedisic S. (2025). Preparation of iron dextran complex and its effect on iron deficiency anemia in rats. Int. J. Biol. Macromol..

[37] Bajrovic I., Schafer S.C., Romanovicz D.K., Croyle M.A. (2020). Novel technology for storage and distribution of live vaccines and other biological medicines at ambient temperature. Sci. Adv..

[38] Si L., Shen Q., Li J., Chen L., Shen J., Xiao X., Bai H., Feng T., Ye A.Y., Li L. (2022). Generation of a live attenuated influenza A vaccine by proteolysis targeting. Nat. Biotechnol..

[39] Voigt E.A., Fuerte-Stone J., Granger B., Archer J., Van Hoeven N. (2021). Live-attenuated RNA hybrid vaccine technology provides single-dose protection against Chikungunya virus. Mol. Ther..

[40] Shang Y., Li L., Zhang T., Luo Q., Yu Q., Zeng Z., Li L., Jia M., Tang G., Fan S. (2022). Quantitative regulation of the thermal stability of enveloped virus vaccines by surface charge engineering to prevent the self-aggregation of attachment glycoproteins. PLoS Pathog..

[41] Du R., Pei F., Kang J., Zhang W., Wang S., Ping W., Ling H., Ge J. (2022). Analysis of the structure and properties of dextran produced by Weissella confusa. Int. J. Biol. Macromol..

[42] Mi X., Blocher McTigue W.C., Joshi P.U., Bunker M.K., Heldt C.L., Perry S.L. (2020). Thermostabilization of viruses via complex coacervation. Biomater. Sci..

[43] Kim A.Y., Kim H., Park S.Y., Park S.H., Kim J.S., Park J.W., Park J.H., Ko Y.J. (2021). Development of a Potent Stabilizer for Long-Term Storage of Foot-and-Mouth Disease Vaccine Antigens. Vaccines.

[44] Aguilera-Garrido A., Del Castillo-Santaella T., Yang Y., Galisteo-Gonzalez F., Galvez-Ruiz M.J., Molina-Bolivar J.A., Holgado-Terriza J.A., Cabrerizo-Vilchez M.A., Maldonado-Valderrama J. (2021). Applications of serum albumins in delivery systems: Differences in interfacial behaviour and interacting abilities with polysaccharides. Adv. Colloid Interface Sci..

[45] Liu Y., Lv W., Shan P., Li D., Wu Y.Q., Wang Y.C., Li Y.Y., Liu Q., Wang J.S., Hao Y.L. (2025). Safety and immunogenicity of an HIV vaccine trial with DNA prime and replicating vaccinia boost. Signal Transduct. Target. Ther..

